# Relationship of Glycated Hemoglobin A1c with All-Cause and Cardiovascular Mortality among Patients with Hypertension

**DOI:** 10.3390/jcm12072615

**Published:** 2023-03-30

**Authors:** Ruixiang Zeng, Yuzhuo Zhang, Junpeng Xu, Yongjie Kong, Jiawei Tan, Liheng Guo, Minzhou Zhang

**Affiliations:** 1 The Second Clinical College of Guangzhou University of Chinese Medicine, Guangzhou 510405, China; ruixiangzeng@gzucm.edu.cn (R.Z.);; 2 Department of Critical Care Medicine, Guangdong Provincial Hospital of Chinese Medicine, Guangzhou 510120, China

**Keywords:** HbA1c, all-cause mortality, cardiovascular mortality, hypertension, NHANES

## Abstract

Both low and high glycated hemoglobin A1c (HbA1c) levels are well-established causal risk factors for all-cause and cardiovascular mortality in the general population and diabetic patients. However, the relationship between HbA1c with all-cause and cardiovascular mortality among patients with hypertension is unclear. We used NHANES data from 1999 to 2014 as the basis for this population-based cohort study. Based on HbA1c levels (HbA1c > 5, HbA1c > 5.5, HbA1c > 6, HbA1c > 6.5, HbA1c > 7%), hypertensive patients were divided into five groups. An analysis of multivariable Cox proportional hazards was conducted based on hazard ratios (HRs) and respective 95% confidence intervals (CIs). The relationship between HbA1c and mortality was further explored using Kaplan–Meier survival curves, restricted cubic spline curves, and subgroup analyses. In addition, 13,508 patients with hypertension (average age 58.55 ± 15.56 years) were included in the present analysis, with 3760 (27.84%) all-cause deaths during a follow-up of 127.69 ± 57.9 months. A U-shaped relationship was found between HbA1c and all-cause and cardiovascular mortality (all *p* for likelihood ratio tests were 0.0001). The threshold value of HbA1c related to the lowest risk for all-cause and cardiovascular mortality was 5.3% and 5.7%, respectively. Below the threshold value, increased HbA1c levels reduced the risk of all-cause mortality (HR 0.68, 95% CI 0.51–0.90, *p* = 0.0078) and cardiovascular mortality (HR 0.77, 95% CI 0.57–1.05, *p* = 0.0969). Inversely, above the threshold value, increased HbA1c levels accelerated the risk of all-cause mortality (HR 1.14, 95% CI 1.11–1.18, *p* < 0.0001) and cardiovascular mortality (HR 1.22, 95% CI 1.16–1.29, *p* < 0.0001). In conclusion, A U-shape relationship was observed between HbA1c and all-cause and cardiovascular mortality among hypertensive patients.

## 1. Introduction

Hypertension continues to be the leading cause of death globally, accounting for 10.4 million deaths per year [1,2]. Due to the similar pathophysiology and mechanisms, such as macrovascular or microvascular disorder, diabetes is not only considered to be one of the most common comorbid conditions with hypertension but also a well-recognized risk factor for hypertension by increasing the risk of mortality [3,4,5]. Glycated hemoglobin A1c (HbA1c) reflects the average blood glucose level in the preceding 8–12 weeks and is suggested by the American Diabetes Association and the World Health Organization for the key diagnostic biomarkers of diabetes [6,7]. Previous studies have reported a correlation between HbA1c values and future all-cause and cardiovascular mortality [8,9,10,11]. Some of them showed that there is a linear relationship, while others considered it as a J- or U-shaped relationship [12,13,14]. Based on these findings, precise HbA1c management is particularly important in both diabetic and non-diabetic populations [2,3,4,10].

However, due to the discrepant research findings of the HbA1c with all-cause and cardiovascular mortality relationships and limited evidence in the hypertension population, there are no published recommendations on the appropriate target HbA1c threshold for the management of hypertension. Meanwhile, the pattern of association between HbA1c and mortality in patients with hypertension is still unclear. Therefore, in the present analysis, we aimed to assess the association between HbA1c variability and the risk of all-cause and cardiovascular mortality in hypertension, as well as to determine a suitable threshold of HbA1c for patients with hypertension.

## 2. Materials and Methods

### 2.1. Study Population

Based on the National Health and Nutrition Examination Survey (NHANES) conducted by the United States National Center for Health Statistics (Centers for Disease Control and Prevention, Atlanta, GA, USA), this population-based cohort study used publicly available data from NHANES from 1999 to 2014. In NHANES, a complex, stratified, multistage, probability sampling method is used to assess the health status of the population. Informed consent was provided by all participants. Further information on the NHANES has been published elsewhere [15,16].

**Figure 1:**
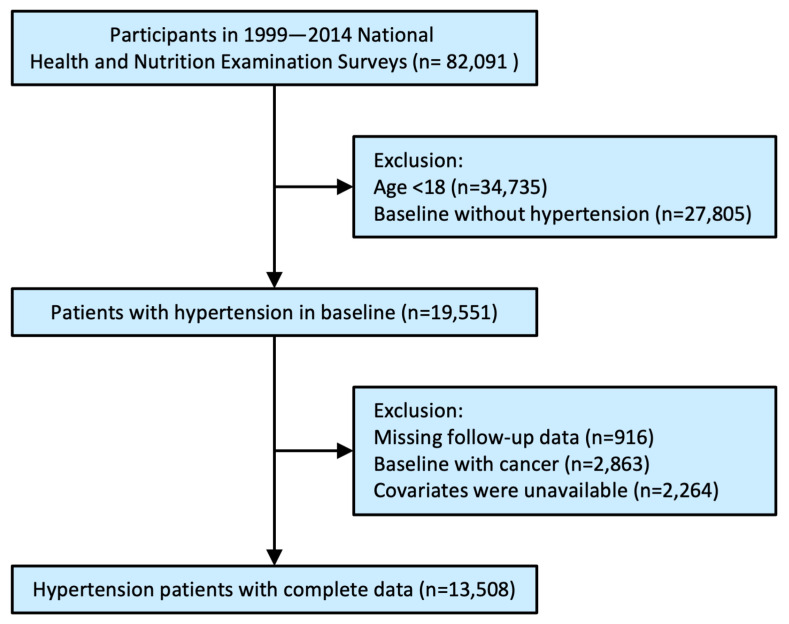
Study flowchart.

Among the 82,091 participants in the primary survey. After excluding participants for age < 18 years old (n = 34,735), baseline without hypertension (n = 27,805), and excluding those missing follow-up data (n = 916), baseline with cancer (n = 2863) and covariates were unavailable (n = 2264). There were 13,508 individuals were enrolled for the analysis (Figure 1).

### 2.2. Demographic Characteristics and Biochemical Covariates

Based on the household interview, demographic variables such as age, gender, race/ethnicity (Mexican American, other Hispanic, non-Hispanic White, non-Hispanic Black, Asian, and other race), and education (Lower than high school, high school, more than high school) were collected.

At baseline, a history of the diseases, such as hypertension, diabetes, coronary heart disease, acute myocardial infarction, chronic heart failure, stroke, and cancer, had been collected by means of standard examinations. Questionnaires were administered by trained health technicians, interviewers, and physicians. The mean blood pressure was calculated by averaging three valid measurements. A diagnosis of diabetes was determined by four criteria as follows: having a history of diabetes, taking anti-diabetes medications, or having FBG > 7.0 mmol/L (126 mg/dL) or HbA1C > 6.5% [17]. Pre-diabetes was defined as having FBG between 5.6 mmol/L (100 mg/dL) and 6.9 mmol/L (125 mg/dL) or HbA1c between 5.7% and 6.4% [18]. Hypertension was defined by one or more criteria as follows: (1) Physician-diagnosed hypertension self-reported by the individual, (2) Antihypertensive drugs taken, (3) systolic and/or diastolic blood pressure (SBP/ DBP) ≥ 140/90 mmHg [19].

In order to conduct laboratory analyses, fasting blood samples were stored at −20 °C and sent to the laboratory for analysis each week. Levels of HbA1c, FBG, creatinine, hemoglobin, and lipid profiles were tested and recorded in authoritative laboratories using standard procedures. Detailed information on laboratory procedures has been published elsewhere [20].

### 2.3. Outcomes

We determined all-cause mortality and cardiovascular mortality in this study. Through 31 December 2018, data from the National Death Index were used to determine the mortality status of the individuals. Based on the ICD-10 codes, this study classified causes of mortality according to causes of mortality. According to ICD-10 codes, cardiovascular mortality falls into four categories: I00-I09, I11, I13, and I20-I51. Deaths due to other causes were censored when cardiovascular mortality was treated as an outcome.

### 2.4. Statistical Analysis

Depending on the data, mean values were presented with standard deviations (SD), median values with interquartile ranges, or frequencies with percentages. An analysis of variance, chi-square test, or Kruskal-Wallis H-test was used to compare the differences between groups based on HbA1c quintile levels. Based on HbA1c categorical data, standardized Kaplan–Meier curves were used for survival analysis. For nonlinear relationships with knots at 5, 35, 65, and 95 percentiles of HbA1c, restricted cubic spline models were used. Two piecewise linear regression models were applied if the relationships were not linear. A threshold value was defined as the value that had the highest likelihood out of all the possible values. By using the logarithmic likelihood ratio test, the results of the one-line and two-line piecewise linear regression models were compared. Based on the log minus log survival curves and the survival times, the proportional hazard assumption was met. A Cox proportional hazards model was used to investigate the association between HbA1c and mortality from all causes. Model 1 did not require any adjustments. Model 2 takes into account the age, gender, and race of the participants. In model 3, we adjusted for age, gender, race, education, body mass index, smoking, coronary heart disease, acute myocardial infarction, chronic heart failure, stroke, creatinine, hemoglobin, triglycerides, total cholesterol, high-density lipoprotein cholesterol, antihypertensive drugs, hypoglycemic agents, aspirin, clopidogrel, and statin. The median value of each categorical of HbA1c, as a continuous variable in the models, was used to test for trends. Furthermore, the subgroup analysis including age (<65 or ≥65 years), gender (male or female), race (White, Black or other race), education (lower than high school, high school, more than high school), BMI (<25 or ≥25 kg/m^2^), smoking (never smoker, current smoker or ex-smoker), diabetes diagnosis (no pre-diabetes, pre-diabetes, diabetes), fasting blood glucose (<5.6, 5.6–6.9, ≥7) coronary heart disease (yes or no), acute myocardial infarction (yes or no), chronic heart failure (yes or no), and stroke (yes or no). A statistically significant value was defined as *p* < 0.05 for all statistical analyses performed using R Version 3.6.1.

## 3. Results

### 3.1. Baseline Characteristics

Finally, 13,508 patients with hypertension (average age 58.55 ± 15.56 years) were included in the present analysis, with 3760 (27.84%) all-cause deaths over a follow-up of 127.69 ± 57.9 months. In Table 1, baseline characteristics are demonstrated according to HbA1c stratification. A significant subgroup difference was observed in age, body mass index, systolic blood pressure, diastolic blood pressure, race, education, smoking, history of the disease, fasting blood glucose, creatinine, hemoglobin, lipid profiles, drug treatment, and all-cause and cardiovascular mortality (all *p* < 0.001), except for gender. 

### 3.2. Relationship between HbA1c and All-Cause and Cardiovascular Mortality

**Figure 2:**
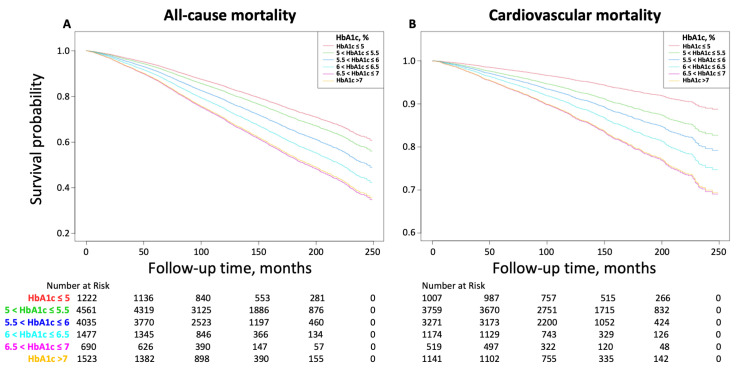
Kaplan–Meier survival curves of HbA1c with all-cause (**A**) and cardiovascular (**B**) mortality. HbA1c, glycated hemoglobin A1c.

As shown in Figure 2, Kaplan–Meier survival curves diverge according to HbA1c level. More risk for both all-cause and cardiovascular mortality was observed when HbA1c ≤ 5% or HbA1c > 7% compared to other groups. 

**Figure 3:**
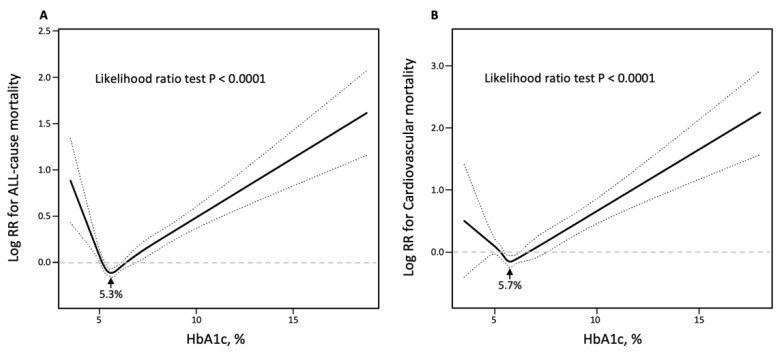
Restricted cubic spine models of HbA1c with all-cause (**A**) and cardiovascular (**B**) mortality in hypertension patients. HbA1c, glycated hemoglobin A1c. Restricted cubic spine models were adjusted for age, gender, race, education, body mass index, smoking, coronary heart disease, acute myocardial infarction, chronic heart failure, stroke, creatinine, hemoglobin, triglycerides, total cholesterol, high-density lipoprotein cholesterol, antihypertensive drugs, hypoglycemic agents, aspirin, clopidogrel, and statin.

Figure 3 illustrated the U-shaped relationship between HbA1c and all-cause and cardiovascular mortality in hypertension patients (All *p* for likelihood ratio test < 0.0001), confirmed by the multivariate-adjusted restrictive cubic curves. Multivariable adjusted analyses showed a threshold value of HbA1c related to the lowest risk of death to all causes was 5.3% and 5.7% for cardiovascular death to all causes. Furthermore, Figure S1 demonstrated the U-shaped relationship of HbA1c with all-cause and cardiovascular mortality only in hypertension patients with pre-diabetes and diabetes, but not in hypertension patients with no pre-diabetes. The threshold value of HbA1c related to the lowest risk for all-cause mortality in hypertension patients with pre-diabetes was 5.9% and with diabetes was 6.5%, and for cardiovascular mortality in hypertension patients with pre-diabetes was 5.9% and with diabetes was 6.7%.

In Table 2, the results of multivariable Cox regression are summarized. In model 3, when HbA1c was treated as a continuous variable, HbA1c corresponded to the hazard ratio (HR) (95% confidence interval, CI) as 1.11 (95% CI 1.08–1.15, *p* < 0.0001) for all-cause mortality, and as 1.17 (95% CI 1.11–1.23, *p* < 0.0001) for cardiovascular mortality. Below the threshold value, increased HbA1c levels reduced the risk of all-cause mortality (HR 0.68, 95% CI 0.51–0.90, *p* = 0.0078) and cardiovascular mortality (HR 0.77, 95% CI 0.57–1.05, *p* = 0.0969). Inversely, above the threshold value, increased HbA1c levels accelerated the risk of all-cause mortality (HR 1.14, 95% CI 1.11–1.18, *p* < 0.0001) and cardiovascular mortality (HR 1.22, 95% CI 1.16–1.29, *p* < 0.0001). When HbA1c was treated as a categorical variable, 5 < HbA1c ≤ 5.5 as a reference, the fully adjusted HRs for all-cause mortality were 1.27 (95% CI 1.11–1.45, *p* = 0.0004), 0.96 (95% CI 0.88–1.05, *p* = 0.3660), 1.07 (95% CI 0.95–1.20 *p* = 0.2622), 1.08 (95% CI 0.93–1.27 *p* = 0.2988), and 1.45 (95% CI 1.27–1.66, *p* < 0.0001) for HbA1c ≤ 5, 5.5 < HbA1c ≤ 6, 6 < HbA1c ≤ 6.5, 6.5 < HbA1c ≤ 7, and HbA1c > 7%, respectively (*p* for trend was < 0.0001). Meanwhile, for cardiovascular mortality, 5.5 < HbA1c ≤ 6 as a reference, the fully adjusted HRs were 1.21 (95% CI 0.92–1.59, *p* = 0.1829), 1.22 (95% CI 1.04 –1.42, *p* = 0.0135), 1.19 (95% CI 0.96–1.46 *p* = 0.1051), 1.23 (95% CI 0.93–1.62, *p* = 0.1510), and 1.85 (95% CI 1.47–2.33, *p* < 0.0001) for HbA1c ≤ 5, 5 < HbA1c ≤ 5.5, 6 < HbA1c ≤ 6.5, 6.5 < HbA1c ≤ 7, HbA1c > 7%, respectively (*p* for trend was 0.0003).

### 3.3. Subgroups Analysis of the Risk of All-Cause and Cardiovascular Mortality

**Figure 4:**
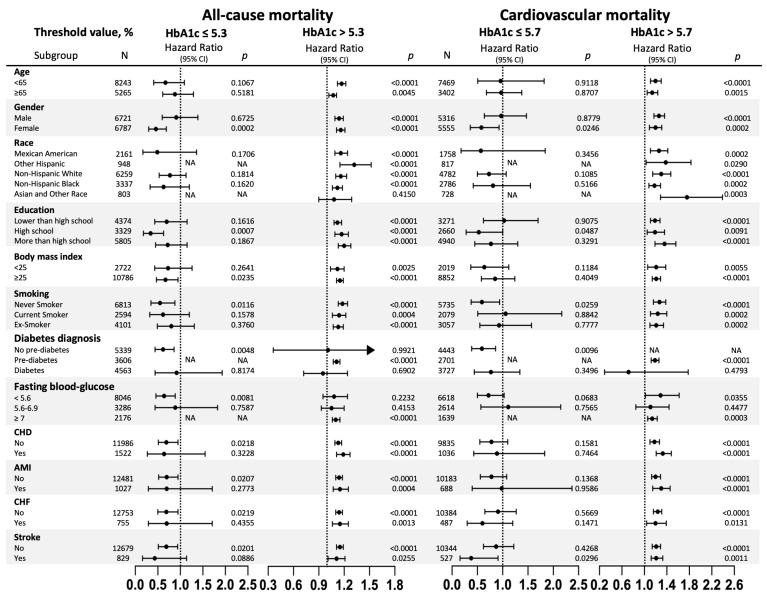
Subgroup analysis. HbA1c, glycated hemoglobin A1c; N, number; HR, hazard ratio; CI, confidence interval; Ex-Smoker, those who previously smoked but had currently stopped; CHD, coronary heart disease; AMI, acute myocardial infarction; CHF, chronic heart failure; NA, not available because of the small sample size. When analyzing a subgroup variable, age, gender, race, education, body mass index, smoking, CHD, AMI, CHF, stroke, creatinine, hemoglobin, triglycerides, total cholesterol, high-density lipoprotein cholesterol, antihypertensive drugs, hypoglycemic agents, aspirin, clopidogrel, and statin were all adjusted except the variable itself, and diabetes was not adjusted in cardiovascular mortality subgroup.

Figure 4 shows the stratified analyses (Detailed data are in Table S1).

All-cause mortality was non-linearly with statistical significance among participants who were female, educated (High school), had body mass indices of ≥25 kg/m^2^, were nonsmokers, without coronary heart disease, without acute myocardial infarction, without chronic heart failure, and without strokes. Meanwhile, in terms of cardiovascular mortality, nonlinear relationships have only been found among participants who were female, nonsmokers, and underwent strokes.

## 4. Discussion

The present study not only determined the baseline HbA1c as a significant predictor of all-cause and cardiovascular mortality for hypertension, in agreement with the earlier study, but also highlighted a U-shaped relationship between HbA1c levels with all-cause and cardiovascular mortality in patients with hypertension. Meanwhile, the threshold value of HbA1c related to the lowest risk for all-cause mortality was 5.3%, and for cardiovascular mortality was 5.7%. According to these findings, patients with hypertension could be guided to control their HbA1c moderately to reduce the risk of mortality, and the optimal target range for the level of HbA1c might be between 5.3% and 5.7%.

Although the level of HbA1C has been shown to be associated with the risk of all-cause and cardiovascular mortality in both adults with and without diabetes, the corresponding relationship on established hypertension has been less well-studied. According to a meta-analysis, the HbA1c levels corresponding to the lowest all-cause mortality and cardiovascular mortality range from 6.0% to 8.0% in patients suffering from diabetes, while 5.0% to 6.0% in non-diabetics [21]. Specifically, there seems to be an increase in all-cause mortality when HbA1c levels are higher than 8.0% or lower than 6.0% in people with diabetes, while the thresholds are 6.0% and 5.0% in non-diabetics [21]. The results of this analysis, especially in the non-diabetic population, are notably similar to those obtained in our study. Further targeting the hypertension population, our study confirmed the U-shaped trend between HbA1c and mortality once again and determined the HbA1c value related to the lowest risk for all-cause and cardiovascular mortality. However, earlier studies have reported a significant linear trend or U-shaped trend between HbA1c and mortality across the general population [22,23]. The discrepancy among the results of these studies is likely to be attributable to the difference in the study population, as well as a constantly updated treatment strategy. In addition, a previous study has demonstrated pre-diabetes might elevate the risk of all-cause and cardiovascular mortality among people with hypertension, but not focused on the threshold level of HbA1C and mortality [18]. Our study also confirmed that the threshold value of HbA1c related to the lowest risk for all-cause mortality in hypertension patients with pre-diabetes was 5.9% and with diabetes, it was 6.5%, and for cardiovascular mortality in hypertension patients with pre-diabetes it was 5.9% and with diabetes, it was 6.7%.

Compared with normotensive individuals, patients with hypertension are often accompanied by hyperglycemia. This comorbid condition, not a coincidence, is initiated by a partly shared pathophysiology between the two states, such as obesity and insulin resistance [5]. However, the long-term elevation of glucose or HbA1c has been shown to activate the advanced glycation end products and receptor for advanced glycation end products (AGEs-RAGE) axis, inflammation, and oxidative stress, which in turn leads to vascular inflammation, endothelial dysfunction, arterial remodeling and atherosclerosis [24,25]. These micro- and macrovascular injuries or dysfunctions could further exacerbate hypertension and target organ (the brain, the heart, the kidneys, the eyes, and the arteries) damage, resulting in increased all-cause and cardiovascular mortality [2]. According to our study, the level of HbA1c is positively correlated with the all-cause and cardiovascular mortality of hypertension when the HbA1c levels exceed the threshold value. Therefore, there is a critical need to control the excessive rise of glucose in people with hypertension.

The influence of relatively low HbA1c on mortality has drawn widespread attention recently. An epidemiological study found that, compared with mid-level HbA1c (5.0% to <5.7%), lower HbA1c (4.0% to <5.0%) is associated with an increased risk of all-cause mortality in non-diabetes [10]. Similarly, we observed more risk for all-cause and cardiovascular mortality in patients with hypertension when HbA1c reduces to levels lower than the threshold value, compared to other ranges. One potential explanation for these findings is the risk factor reversal, a phenomenon caused by the shared role of protein-energy malnutrition and inflammatory disorders [8,26]. Meanwhile, when the glucose is too low, hemodynamics could change to maintain a sufficient glucose supply for critical organs, resulting in an increase in heart rate, myocardial contractility, and peripheral systolic blood pressure, thereby increasing the risk of major cardiovascular events [27]. In addition, hypoglycemia could lead to QT interval prolongation, which may increase the risk of cardiac arrhythmia and contribute to cardiovascular mortality [28]. 

Hence, an overly low level of glucose is not beneficial and should be prevented for long-term survival, especially in patients with hypertension.

With its relatively large sample size and long-term follow-up, the present study contributes to the literature and provides further evidence of the U-shaped relationship between HbA1C and mortality. Regarding clinical importance, our novel findings are conducive to understanding the risk stratification of HbA1c and remind us that when developing therapeutic strategies for patients with hypertension, attention should be paid to assessing the absolute risk of HbA1c, rather than starting treatment based solely on changes of cardiovascular indexes.

There are, however, still some limitations to the present study. First, at baseline, only one measurement of serum HbA1c concentration is available, resulting in potential bias and failure to assess HbA1c’s effects. Second, although we adjusted for confounding variables, residual confounding from unknown or unmeasured factors remains possible. Finally, we use data from the NHANES study conducted by the United States National Center, rendering the conclusion not easy to extrapolate to the population in other regions.

## 5. Conclusions

This is the first report on the U-shaped associations between HbA1c and the risk of mortality among patients with hypertension. Judging from the present result, the optimal target range for the level of HbA1c might be between 5.3% and 5.7%, implying an increased risk in all-cause and cardiovascular mortality of hypertension at both higher and lower levels of HbA1c. The issue with the association between hypertension and HbA1c suggests the necessity of introducing HbA1c monitoring into the management of hypertension and making treatment strategies for patients with hypertension in a multidisciplinary fashion. 

## Figures and Tables

**Table 1:** Baseline characteristics according to HbA1c quintiles.

	Total	HbA1c ≤ 5	5 < HbA1c ≤ 5.5	5.5 < HbA1c ≤ 6	6 < HbA1c ≤ 6.5	6.5 < HbA1c ≤ 7	HbA1c > 7	*p*
N	13,508	1222	4561	4035	1477	690	1523	
Age, years	58.55 ± 15.56	48.91 ± 17.38	55.77 ± 16.65	60.91 ± 14.38	63.97 ± 12.54	63.99 ± 12.18	60.67 ± 12.15	<0.001
Male, N (%)	6787 (50.24)	609 (49.84)	2287 (50.14)	2055 (50.93)	778 (52.67)	323 (46.81)	735 (48.26)	0.075
Body mass index, kg/m^2^	30.45 ± 6.91	28.51 ± 6.52	29.23 ± 6.31	30.59 ± 6.93	32.00 ± 7.06	32.82 ± 7.47	32.71 ± 7.21	<0.001
Systolic blood pressure, mmHg	135.80 ± 20.78	132.32 ± 20.94	135.38 ± 20.46	136.71 ± 20.92	136.13 ± 20.15	134.84 ± 19.71	137.53 ± 21.91	<0.001
Diastolic blood pressure, mmHg	73.53 ± 14.05	75.53 ± 14.10	75.24 ± 13.71	73.53 ± 13.96	71.17 ± 13.79	69.39 ± 14.50	70.94 ± 14.17	<0.001
Race, N (%)								<0.001
Mexican American	2161 (16.00)	132 (10.80)	676 (14.82)	621 (15.39)	216 (14.62)	126 (18.26)	390 (25.61)	
Other Hispanic	948 (7.02)	64 (5.24)	273 (5.99)	291 (7.21)	136 (9.21)	57 (8.26)	127 (8.34)	
Non-Hispanic White	6259 (46.34)	680 (55.65)	2528 (55.43)	1811 (44.88)	536 (36.29)	252 (36.52)	452 (29.68)	
Non-Hispanic Black	3337 (24.70)	285 (23.32)	867 (19.01)	1046 (25.92)	486 (32.90)	206 (29.86)	447 (29.35)	
Asian and other Race	803 (5.94)	61 (4.99)	217 (4.76)	266 (6.59)	103 (6.97)	49 (7.10)	107 (7.03)	
Education, N (%)								<0.001
Lower than high school	4374 (32.38)	274 (22.42)	1310 (28.72)	1304 (32.32)	532 (36.02)	273 (39.57)	681 (44.71)	
High school	3329 (24.64)	285 (23.32)	1102 (24.16)	1053 (26.10)	400 (27.08)	168 (24.35)	321 (21.08)	
More than high school	5805 (42.97)	663 (54.26)	2149 (47.12)	1678 (41.59)	545 (36.90)	249 (36.09)	521 (34.21)	
Smoking, N (%)								<0.001
Never Smoker	6813 (50.44)	620 (50.74)	2306 (50.56)	2051 (50.83)	716 (48.48)	339 (49.13)	781 (51.28)	
Current Smoker	2594 (19.20)	286 (23.40)	924 (20.26)	771 (19.11)	249 (16.86)	109 (15.80)	255 (16.74)	
Ex-Smoker	4101 (30.36)	316 (25.86)	1331 (29.18)	1213 (30.06)	512 (34.66)	242 (35.07)	487 (31.98)	
Diabetes diagnosis, N (%)								<0.001
No pre-diabetes	5339 (39.52)	1029 (84.21)	3572 (78.32)	738 (18.29)	0 (0.00)	0 (0.00)	0 (0.00)	
Pre-diabetes	4563 (33.78)	150 (12.27)	819 (17.96)	2812 (69.69)	782 (52.95)	0 (0.00)	0 (0.00)	
Diabetes	3606 (26.70)	43 (3.52)	170 (3.73)	485 (12.02)	695 (47.05)	690 (100.00)	1523 (100.00)	
Coronary heart disease, N (%)	1522 (11.27)	64 (5.24)	406 (8.90)	467 (11.57)	206 (13.95)	116 (16.81)	263 (17.27)	<0.001
Acute myocardial infarction, N (%)	1027 (7.60)	36 (2.95)	277 (6.07)	308 (7.63)	145 (9.82)	84 (12.17)	177 (11.62)	<0.001
Chronic heart failure, N (%)	755 (5.59)	30 (2.45)	194 (4.25)	208 (5.15)	111 (7.52)	74 (10.72)	138 (9.06)	<0.001
Stroke, N (%)	829 (6.14)	58 (4.75)	224 (4.91)	252 (6.25)	115 (7.79)	54 (7.83)	126 (8.27)	<0.001
HbA1c, %	5.98 ± 1.24	4.83 ± 0.22	5.33 ± 0.13	5.77 ± 0.14	6.26 ± 0.14	6.78 ± 0.14	8.76 ± 1.71	<0.001
Fasting blood glucose, mmol/L	6.09 ± 2.54	5.00 ± 0.72	5.17 ± 0.75	5.50 ± 0.99	6.18 ± 1.41	7.12 ± 2.04	10.71 ± 4.73	<0.001
Creatinine, mg/dL	85.73 ± 54.06	86.58 ± 76.68	83.23 ± 56.50	84.71 ± 35.56	87.07 ± 40.57	93.41 ± 77.70	90.48 ± 62.48	<0.001
Hemoglobin, g/L	14.11 ± 1.55	14.10 ± 1.78	14.32 ± 1.51	14.11 ± 1.49	13.85 ± 1.52	13.79 ± 1.59	13.86 ± 1.58	<0.001
Triglyceride, mg/dL	134.0 (90.0–202.0)	116.0 (77.0–172.0)	123.0 (84.0–185.0)	134.0 (92.0–193.0)	144.0 (99.0–212.0)	154.0 (104.3–233.8)	173.0 (114.5–266.5)	<0.001
Total cholesterol, mg/dL	200.13 ± 43.65	195.61 ± 40.24	203.31 ± 41.72	203.78 ± 43.61	193.48 ± 44.09	189.08 ± 46.87	196.06 ± 47.72	<0.001
HDL-C, mg/dL	51.95 ± 15.92	55.38 ± 17.81	54.08 ± 16.84	52.32 ± 15.46	49.24 ± 13.76	47.65 ± 14.11	46.38 ± 12.96	<0.001
Antihypertensive drugs, N (%)	8015 (59.34)	508 (41.57)	2212 (48.50)	2506 (62.11)	1091 (73.87)	565 (81.88)	1133 (74.39)	<0.001
Hypoglycemic agents, N (%)	2423 (17.94)	22 (1.80)	81 (1.78)	312 (7.73)	434 (29.38)	421 (61.01)	1153 (75.71)	<0.001
Aspirin, N (%)	322 (2.38)	11 (0.90)	50 (1.10)	95 (2.35)	62 (4.20)	27 (3.91)	77 (5.06)	<0.001
Clopidogrel, N (%)	438 (3.24)	19 (1.55)	104 (2.28)	128 (3.17)	66 (4.47)	37 (5.36)	84 (5.52)	<0.001
Statin, N (%)	3742 (27.70)	129 (10.56)	864 (18.94)	1116 (27.66)	628 (42.52)	326 (47.25)	679 (44.58)	<0.001
All-cause mortality, N (%)	3760 (27.84)	278 (22.75)	1154 (25.30)	1089 (26.99)	440 (29.79)	245 (35.51)	554 (36.38)	<0.001
Cardiovascular mortality, N (%)	1123 (10.33)	63 (6.26)	352 (9.36)	325 (9.94)	137 (11.67)	74 (14.26)	172 (15.07)	<0.001

HbA1c, glycated hemoglobin A1c; N, number; Ex-Smoker, those who previously smoked but had currently stopped; HDL-C, high-density lipoprotein cholesterol. Values are expressed as the mean ± SD, the median with interquartile range or n (%).

**Table 2:** Multivariate Cox regression analysis of HbA1c with all-cause and cardiovascular mortality.

	Model 1 HR (95% CI), *p*	Model 2 HR (95% CI), *p*	Model 3 HR (95% CI), *p*
**All-cause mortality**			
HbA1c	1.13 (1.11, 1.16), <0.0001	1.15 (1.12, 1.18), <0.0001	1.11 (1.08, 1.15), <0.0001
**HbA1c threshold value**			
≤threshold value 5.3	1.04 (0.77, 1.40), 0.8085	0.53 (0.40, 0.70), <0.0001	0.68 (0.51, 0.90), 0.0078
>threshold value 5.3	1.10 (1.08, 1.13), <0.0001	1.19 (1.16, 1.22), <0.0001	1.14 (1.11, 1.18), <0.0001
**HbA1c categorical**			
HbA1c ≤ 5	0.86 (0.76, 0.98), 0.0256	1.32 (1.16, 1.51), <0.0001	1.27 (1.11, 1.45), 0.0004
5 < HbA1c ≤ 5.5	Reference	Reference	Reference
5.5 < HbA1c ≤ 6	1.23 (1.14, 1.34), <0.0001	0.98 (0.90, 1.06), 0.6200	0.96 (0.88, 1.05), 0.3660
6 < HbA1c ≤ 6.5	1.49 (1.33, 1.66), <0.0001	1.13 (1.01, 1.27), 0.0294	1.07 (0.95, 1.20), 0.2622
6.5 < HbA1c ≤ 7	1.82 (1.59, 2.09), <0.0001	1.31 (1.14, 1.51), 0.0001	1.08 (0.93, 1.27), 0.2988
HbA1c > 7	1.79 (1.61, 1.98), <0.0001	1.73 (1.56, 1.92), <0.0001	1.45 (1.27, 1.66), <0.0001
*p* for trend	<0.0001	<0.0001	<0.0001
**Cardiovascular mortality**			
HbA1c	1.17 (1.13, 1.21), <0.0001	1.20 (1.15, 1.25), <0.0001	1.17 (1.11, 1.23), <0.0001
**HbA1c threshold value**			
≤threshold value 5.7	1.80 (1.33, 2.45), 0.0002	0.76 (0.56, 1.03), 0.0776	0.77 (0.57, 1.05), 0.0969
>threshold value 5.7	1.09 (1.04, 1.14), 0.0005	1.24 (1.18, 1.30), <0.0001	1.22 (1.16, 1.29), <0.0001
**HbA1c categorical**			
HbA1c ≤ 5	0.51 (0.39, 0.67), <0.0001	1.17 (0.89, 1.53), 0.2681	1.21 (0.92, 1.59), 0.1829
5 < HbA1c ≤ 5.5	0.81 (0.70, 0.95), 0.0078	1.13 (0.97, 1.32), 0.1080	1.22 (1.04, 1.42), 0.0135
5.5 < HbA1c ≤ 6	Reference	Reference	Reference
6 < HbA1c ≤ 6.5	1.25 (1.02, 1.53), 0.0290	1.24 (1.01, 1.51), 0.0386	1.19 (0.96, 1.46), 0.1051
6.5 < HbA1c ≤ 7	1.59 (1.23, 2.05), 0.0003	1.40 (1.09, 1.81), 0.0089	1.23 (0.93, 1.62), 0.1510
HbA1c > 7	1.57 (1.30, 1.89), <0.0001	2.05 (1.70, 2.48), <0.0001	1.85 (1.47, 2.33), <0.0001
*p* for trend	<0.0001	<0.0001	0.0003

HbA1c, glycated hemoglobin A1c; HR, hazard ratio; CI, confidence interval. Model 1: no adjustment; Model 2: adjusted for age, gender, and race; Model 3: adjusted for age, gender, race, education, body mass index, smoking, coronary heart disease, acute myocardial infarction, chronic heart failure, stroke, creatinine, hemoglobin, triglycerides, total cholesterol, high-density lipoprotein cholesterol, antihypertensive drugs, hypoglycemic agents, aspirin, clopidogrel, and statin.

## Data Availability

https://www.cdc.gov/nchs/nhanes/index.htm (accessed on 6 November 2022).

## Supplementary Materials

The following supporting information can be downloaded at: https://www.mdpi.com/article/10.3390/jcm12072615/s1, Figure S1: Restricted cubic spine models of HbA1c with all-cause and cardiovascular mortality in hypertension patients with no pre-diabetes (A1,A2), pre-diabetes (B1,B2) and diabetes (C1,C2). HbA1c, glycated hemoglobin A1c. Restricted cubic spine models were adjusted for adjusted for age, gender, race, education, body mass index, smoking, coronary heart disease, acute myocardial infarction, chronic heart failure, stroke, creatinine, hemoglobin, triglycerides, total cholesterol, high-density lipoprotein cholesterol, antihypertensive drugs, hypoglycemic agents, aspirin, clopidogrel, and statin; Table S1: Data for Figure 4 (Subgroup analysis).
