# Supplementary material for: Relationship of Glycated Hemoglobin A1c with All-Cause and Cardiovascular Mortality among Patients with Hypertension
Source: J Clin Med. 2023 Mar 30;12(7):2615. doi: 10.3390/jcm12072615 (PMC10095266; doi:10.3390/jcm12072615)
Supplement: Supplementary file 1 [file jcm-12-02615-s001.zip › jcm-2170186-supplementary.pdf]

Table S1. Data for Figure 4 (Subgroup analysis)

	All-cause mortality, HR (95% CI) , *p*			Cardiovascular mortality, HR (95% CI), *p*		
**Threshold value, %**	**N**	**HbA1c ≤ 5.3**	**HbA1c > 5.3**	**N**	**HbA1c ≤ 5.7**	**HbA1c > 5.7**
**Age**						
<65	8243	0.67 (0.41, 1.09) ,0.1067	1.17 (1.12, 1.22) ,<0.0001	7469	0.96 (0.51, 1.82), 0.9118	1.20 (1.11, 1.30) ,<0.0001
≥65	5265	0.88 (0.61, 1.29) ,0.5181	1.07 (1.02, 1.11), 0.0045	3402	0.97 (0.68, 1.38), 0.8707	1.14 (1.05, 1.24) ,0.0015
**Gender**						
Male	6721	0.91 (0.60, 1.39) ,0.6725	1.14 (1.09, 1.19) ,<0.0001	5316	0.97 (0.64, 1.47) ,0.8779	1.26 (1.17, 1.36) ,<0.0001
Female	6787	0.46 (0.30, 0.69) ,0.0002	1.16 (1.11, 1.21), <0.0001	5555	0.58 (0.36, 0.93) ,0.0246	1.20 (1.09, 1.31) ,0.0002
**Race**						
Mexican American	2161	0.49 (0.17, 1.36), 0.1706	1.16 (1.09, 1.24), <0.0001	1758	0.57 (0.18, 1.84) ,0.3456	1.26 (1.11, 1.42) ,0.0002
Other Hispanic	948	NA	1.32 (1.15, 1.52), <0.0001	817	NA	1.38 (1.03, 1.83) ,0.0290
Non-Hispanic White	6259	0.77 (0.53, 1.13) ,0.1814	1.16 (1.10, 1.23) ,<0.0001	4782	0.73 (0.50, 1.07) ,0.1085	1.30 (1.15, 1.47), <0.0001
Non-Hispanic Black	3337	0.63 (0.33, 1.20) ,0.1620	1.12 (1.06, 1.18), <0.0001	2786	0.81 (0.42, 1.55) ,0.5166	1.18 (1.08, 1.29) ,0.0002
Asian and Other Race	803	NA	1.08 (0.90, 1.29) ,0.4150	728	NA	1.76 (1.29, 2.39) ,0.0003
**Education**						
Lower than high school	4374	0.70 (0.43, 1.15) ,0.1616	1.12 (1.08, 1.17), <0.0001	3271	1.03 (0.62, 1.70) ,0.9075	1.19 (1.11, 1.28) ,<0.0001
High school	3329	0.34 (0.18, 0.63) ,0.0007	1.17 (1.09, 1.25), <0.0001	2660	0.53 (0.28, 1.00) ,0.0487	1.19 (1.05, 1.36) ,0.0091
More than high school	5805	0.72 (0.45, 1.17) ,0.1867	1.20 (1.13, 1.28), <0.0001	4940	0.77 (0.45, 1.30) ,0.3291	1.36 (1.19, 1.56) ,<0.0001
**Body mass index**						
<25	2722	0.73 (0.43, 1.26) ,0.2641	1.12 (1.04, 1.20) ,0.0025	2019	0.64 (0.37, 1.12) ,0.1184	1.21 (1.06, 1.38) ,0.0055
≥25	10786	0.67 (0.47, 0.95) ,0.0235	1.15 (1.11, 1.19) ,<0.0001	8852	0.85 (0.58, 1.24) ,0.4049	1.21 (1.14, 1.29) ,<0.0001
**Smoking**						
Never Smoker	6813	0.55 (0.35, 0.88) ,0.0116	1.18 (1.13, 1.24) ,<0.0001	5735	0.59 (0.38, 0.94) ,0.0259	1.27 (1.16, 1.38), <0.0001
Current Smoker	2594	0.62 (0.32, 1.20) ,0.1578	1.14 (1.06, 1.22) ,0.0004	2079	1.06 (0.51, 2.17) ,0.8842	1.24 (1.11, 1.40) ,0.0002
Ex-Smoker	4101	0.80 (0.49, 1.31) ,0.3760	1.13 (1.07, 1.19) ,<0.0001	3057	0.93 (0.55, 1.57) ,0.7777	1.21 (1.10, 1.34) ,0.0002

**Diabetes diagnosis**						
No pre-diabetes	5339	0.62 (0.44, 0.86), 0.0048	1.01 (0.36, 2.79), 0.9921	4443	0.59 (0.39, 0.88), 0.0096	NA
Pre-diabetes	3606	NA	1.11 (1.07, 1.15) ,<0.0001	2701	NA	1.19 (1.12, 1.26), <0.0001
Diabetes	4563	0.92 (0.44, 1.92), 0.8174	0.95 (0.72, 1.24) ,0.6902	3727	0.77 (0.44, 1.34) ,0.3496	0.72 (0.29, 1.78) ,0.4793
**Fasting blood-glucose**						
<5.6	8046	0.64 (0.46, 0.89) ,0.0081	1.08 (0.95, 1.24), 0.2232	6618	0.72 (0.50, 1.03) ,0.0683	1.29 (1.02, 1.62) ,0.0355
5.6-6.9	3286	0.89 (0.44, 1.83) ,0.7587	1.05 (0.93, 1.20) ,0.4153	2614	1.11 (0.57, 2.15) ,0.7565	1.11 (0.85, 1.44) ,0.4477
≥7	2176	NA	1.10 (1.06, 1.15), <0.0001	1639	NA	1.14 (1.06, 1.23) ,0.0003
**Coronary heart disease**						
NO	11986	0.69 (0.51, 0.95) I,0.0218	1.13 (1.09, 1.17) ,<0.0001	9835	0.78 (0.55, 1.10) ,0.1581	1.18 (1.10, 1.27) ,<0.0001
YES	1522	0.64 (0.27, 1.55) ,0.3228	1.19 (1.11, 1.27) ,<0.0001	1036	0.89 (0.43, 1.83) ,0.7464	1.33 (1.21, 1.48) ,<0.0001
**Acute myocardial infarction**						
NO	12481	0.70 (0.52, 0.95) ,0.0207	1.14 (1.10, 1.18) ,<0.0001	10183	0.78 (0.56, 1.08) ,0.1368	1.20 (1.13, 1.29) ,<0.0001
YES	1027	0.52 (0.16, 1.68) ,0.2773	1.15 (1.07, 1.25) ,0.0004	688	0.98 (0.40, 2.37) ,0.9586	1.30 (1.15, 1.46) ,<0.0001
**Chronic heart failure**						
NO	12753	0.69 (0.50, 0.95) ,0.0219	1.14 (1.10, 1.18) ,<0.0001	10384	0.91 (0.65, 1.27) ,0.5669	1.24 (1.16, 1.31) ,<0.0001
YES	755	0.70 (0.29, 1.71) ,0.4355	1.15 (1.06, 1.25) ,0.0013	487	0.60 (0.30, 1.20) ,0.1471	1.20 (1.04, 1.39) ,0.0131
**Stroke**						
NO	12679	0.69 (0.51, 0.94) ,0.0201	1.15 (1.11, 1.19), <0.0001	10344	0.87 (0.62, 1.22) ,0.4268	1.21 (1.14, 1.29) ,<0.0001
YES	829	0.43 (0.16, 1.14) ,0.0886	1.11 (1.01, 1.21), 0.0255	527	0.38 (0.16, 0.91) ,0.0296	1.33 (1.12, 1.58) ,0.0011

HbA1c, glycated hemoglobin A1c; N, number; HR, hazard ratio; CI, confidence interval; NA, not available because of the small sample size. Ex-Smoker, those who previously smoked but had currently stopped; When analyzing a subgroup variable, age, gender, race, education, body mass index, smoking, coronary heart disease, acute myocardial infarction, chronic heart failure, stroke, creatinine, hemoglobin, triglycerides, total cholesterol, high-density lipoprotein cholesterol, antihypertensive drugs, hypoglycemic agents, aspirin, clopidogrel, and statin were all adjusted except the variable itself, and diabetes was not adjusted in cardiovascular mortality subgroup.

Figure S1

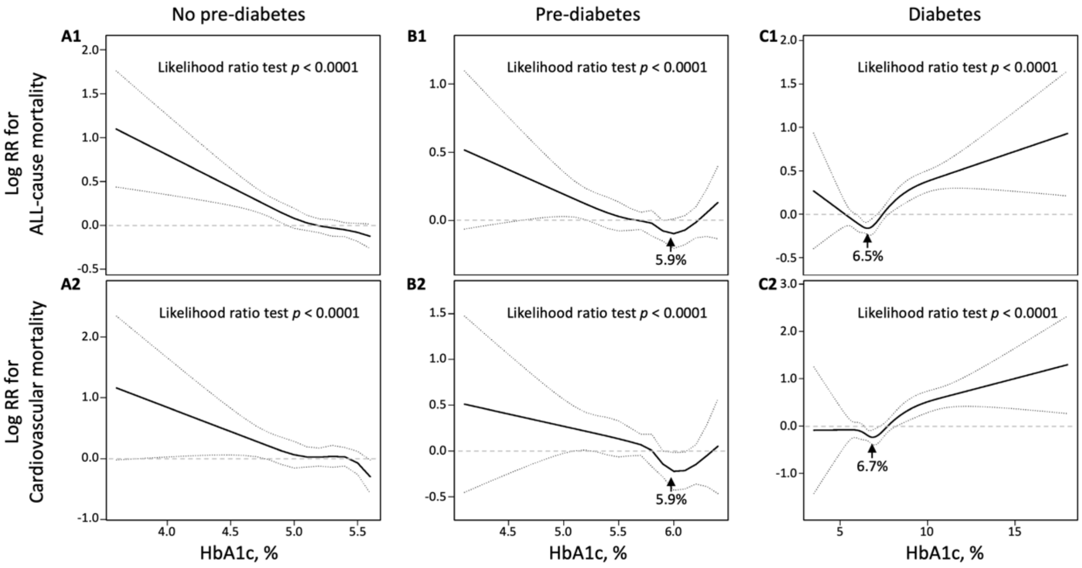

Figure S1. Restricted cubic spine models of HbA1c with all-cause and cardiovascular mortality in hypertension patients with no pre-diabetes (A1&A2), pre-diabetes (B1&B2) and diabetes (C1&C2). HbA1c, glycated hemoglobin A1c. Restricted cubic spine models were adjusted for adjusted for age, gender, race, education, body mass index, smoking, coronary heart disease, acute myocardial infarction, chronic heart failure, stroke, creatinine, hemoglobin, triglycerides, total cholesterol, high-density lipoprotein cholesterol, antihypertensive drugs, hypoglycemic agents, aspirin, clopidogrel, and statin.
